# Late-onset hearing loss was not observed among preschool- aged children with prenatal Zika virus exposure: An analysis of the Microcephaly Epidemic Research Group Pediatric Cohort (2015–2019)

**DOI:** 10.1371/journal.pntd.0013033

**Published:** 2025-04-25

**Authors:** Danielle Seabra Ramos, Lilian Ferreira Muniz, Mariana de Carvalho Leal, Demócrito de Barros Miranda Filho, Ricardo Arraes de Alencar Ximenes, Elizabeth Brickley, Thalia Velho Barreto de Araujo, Celina Maria Turchi Martelli, Silvio da Silva Caldas Neto

**Affiliations:** 1 Escola de Saúde e Ciências da vida, Universidade Católica de Pernambuco, Recife, Brasil; 2 Faculdade Pernambucana de Saúde, Recife, Brasil; 3 Programa de Pós-graduacão de Saude da Comunicacão Humana, Universidade Federal de Pernambuco, Recife, Brasil; 4 Programa de Pós-Graduação em Ciências da Saúde FCM/Universidade de Pernambuco, Recife, Brasil; 5 Departamento de Medicina Tropical, Universidade Federal de Pernambuco, Recife, Pernambuco, Brasil; 6 Universidade de Brasilia, Brasilia, Brasil; 7 Universidade Gama Filho, Rio de Janeiro, Brasil; 8 London School of Hygiene & Tropical Medicine, London, United Kingdom; 9 Departamento de Medicina Social, Universidade Federal de Pernambuco, Recife, Brasil; 10 Centro de Pesquisa Aggeu Magalhães, Fiocruz PE, Ministerio da Saude, Recife, Brasil; 11 Departamento de Cirurgia, Universidade Federal de Pernambuco, Recife, Brasil; Australian Red Cross Lifeblood, AUSTRALIA

## Abstract

Zika virus (ZIKV) infection during pregnancy can lead to Congenital Zika Syndrome (CZS), with significant impacts on early childhood development. This study aimed to analyze the natural history of hearing loss in children with CZS during their first four years of life. Data were collected from the Microcephaly Epidemic Research Group Pediatric Cohort in Pernambuco, Brazil. We investigated whether children with prenatal ZIKV exposure could develop late-onset hearing loss and whether those with preserved auditory thresholds at birth might exhibit deficits in functional hearing or primary language development.The study included children with suspected or confirmed prenatal ZIKV exposure who had normal neonatal hearing screenings. Follow-up assessments included auditory brainstem response testing and behavioral observational audiometry. Children were evaluated every six months up to 24 months of age and annually thereafter until 48 months. Results indicated that late-onset sensorineural hearing loss (SNHL) was not observed in children with prenatal ZIKV exposure and normal neonatal hearing screening. The hearing losses identified were transient and typically related to middle ear effusion. Therefore, our findings reinforce that congenital SNHL associated with ZIKV exposure predominantly manifests at birth, with no evidence supporting its late onset in the first years of life.

## Author summary

Our study investigates the auditory outcomes of prenatal Zika virus (ZIKV) exposure, with a specific focus on hearing loss in children exposed to ZIKV during fetal life. Over a longitudinal follow-up of four years, this study evaluated children from a well-characteri cohort in Pernambuco, Brazil, who had normal neonatal hearing screening through auditory brainstem response (ABR) testing. We did not find evidence of late-onset sensorineural hearing loss (SNHL) among children with prenatal ZIKV exposure. Transient hearing impairments observed were attributed to middle ear conditions, such as effusions, rather than to persistent or progressive sensorineural damage. These results emphasize that children exposed to ZIKV during pregnancy, particularly those without microcephaly, are not at increased risk of long-term auditory deficits, providing evidence for refining clinical follow-up strategies.

## Background

Zika virus (ZIKV) has a marked neurotropism, and infections during pregnancy may result in Congenital Zika Syndrome (CZS), with deleterious consequences for children’s development in the first years of life. In addition to damaging brain tissue, congenital ZIKV infections may compromise children’s peripheral auditory and visual systems, impairing their social interactions and making stimulation and rehabilitation even more challenging [1,2,3,4,5,6].

A 2015–2016 cross-sectional study by Leal, et al., of 69 infants with ZIKV-related microcephaly estimated the prevalence of sensorineural hearing loss (SNHL) diagnosed using frequency-specific auditory brainstem responses (ABR) to be 5.8% [4]. Similarly, the 2023 individual participant data meta-analysis of the Zika Brazilian Cohorts-Consortium (ZBC-Consortium) found the frequency of concomitant failure for ABR and otoacoustic emission (OAE) to be 6.4% among children, at birth or in the first evaluation (aged in average 6,5 months), with reverse transcription--polymerase chain reaction (RT-PCR)-confirmed prenatal ZIKV exposure [7]. Previous studies have reported varied prevalence rates of sensorineural hearing loss (SNHL) in children with prenatal ZIKV exposure, ranging from 5.1% to 6.4%, depending on the diagnostic methods and study populations. While some research identified an increased risk of SNHL, particularly in children with microcephaly, the trajectory of hearing outcomes in normocephalic children remains unclear. Additionally, limitations in prior studies, such as the use of screening tools that do not detect mild hearing loss and the absence of longitudinal data, hinder definitive conclusions about late-onset SNHL in this population. This study addresses these gaps by providing long-term auditory follow-up of children with prenatal ZIKV exposure [8].

Preliminary findings from studies evaluating children with laboratory evidence of prenatal ZIKV infection and microcephaly during the first three years of life found an incidence of SNHL diagnosed through neonatal hearing screening and audiological diagnosis of 9.3%, a frequency higher than that observed in the general population, where this alteration is present in approximately 1.7/1,000 live births. Importantly, this data provides no evidence of progressive or late onset SNHL [9]. This phenomenon is observed in other congenital infections, such as cytomegalovirus, where all degrees of hearing loss can occur in both symptomatic and asymptomatic cases. Notably, late-onset hearing loss is reported in 9–68% of cases, and progressive hearing loss over time is observed in 7–71% of cases of cytomegalovirus prenatal exposure [10,11]. Thus, we hypothesize that late onset hearing loss could occur in children with prenatal ZIKV exposure, despite normal neonatal hearing screening, as it happens with other congenital infections like cytomegalovirus, and deficits in functional hearing and language development also could occur in these children life due to motor impairments that may limit their ability to seek sound sources and fully engage with their environment.

A recent meta-analysis involving normocephalic children with prenatal ZIKV exposure exhibited a higher prevalence of neurodevelopmental delays, particularly in language (29.7%), but there was heterogeneity across studies and vulnerability factors such as parental education levels, limited access to prenatal care, and barriers to early diagnostics and interventions, were potential confounding factors in this analisis of language delays [12]. Two other more recent studies, however, did not find differences in the neurodevelopment between normocephalic children exposed and unexposed in utero to ZIKV [13,14]. In another study by our group focusing on behavioral response to sound and the development of communicative skills, we evaluated 88 children with laboratory evidence of prenatal ZIKV infection and observed communication delays in 87.5% of the children despite normal peripheral hearing according automatic ABR evaluation. In this study sample, most children had microcephaly and lacked cervical motor control, a clinical feature that was statistically associated with a greater delay in auditory and communication skills [15]. However it is possible that behavioral and motor impairments could contribute to misclassifications in evaluating deficits in functional hearing and language development.

Using data collected from children with prenatal ZIKV exposure followed-up prospectively over the first four years of life as part of the Microcephaly Epidemic Research Group Pediatric Cohort in Pernambuco [16], Brazil, this study aimed to better understand the natural history of hearing loss in children with prenatal ZIKV exposure during the early life course. Specifically, we aimed to clarify whether (i) hearing loss among children with CZS may occur with a late-onset and (ii) if children with with prenatal ZIKV exposure who have preserved auditory thresholds could present with deficits in functional hearing and language development.

## Method

### Ethics statement

The project was approved by the Ethics Committee of Hospital Universitário Oswaldo Cruz (CAAE: 52803316.8.0000.5192) and followed the ethical procedures recommended for this type of study by Resolution MS/CNS 466/2012. Written free and informed consent was provided by all mothers or legal guardians of the children.

The present study is an analysis of children with suspected or laboratory- confirmed prenatal ZIKV exposure participating in the Microcephaly Epidemic Research Group Pediatric Cohort (MERG-PC) based in Pernambuco, Brazil [11]. Study participants were followed-up throughout the first four years of life and included children who had previously passed the neonatal hearing screening (NHS) performed in the first year of life. Children who failed the NHS were retested, and those who failed the cABR retest were referred for audiological diagnosis and excluded from the study in case of identified sensorineural hearing loss.

### Study population

The MERG-PC includes children born between 2015 and 2017, during the Zika- related microcephaly epidemic in Pernambuco. As previously described (REF – Cohort Profile), participants were recruited from:

i) Children whose mothers had rash and laboratory evidence of ZIKV infection during pregnancy, regardless of whether the children had symptoms of CZS. As previously described [18,19], laboratory evidence was established through maternal tests performed on up to three serological samples, the first collected within five days of the onset of the rash, the second at least 14 days after notification and the last sample collected after delivery. Maternal sera were evaluated for the presence of ZIKV ribonucleic acid (RNA) by quantitative reverse-transcription polymerase chain reaction (qRT-PCR) and for the detection of ZIKV-specific immunoglobulin (Ig)M antibodies by IgM capture enzyme-linked immunosorbent assay (MAC-ELISA) and anti-nonstructural protein 1 (NS1) IgG3 antibodies specific for ZIKV [16].
ii) Children with microcephaly at birth and laboratorial confirmed prenatal ZIKV exposure through qRT-PCR in a cerebrospinal fluid sample, serologic IgM antibodies detection using MAC-ELISA or based on the criteria of França, et al (2016) [20,21]. Microcephaly was defined by a head circumference (HC) two or more standard deviations below the mean for age and sex [17,18,19,20]. While for term infants born at gestational age of at least 37 weeks, head circumference was assessed using the World Health Organization (WHO) Anthro software and classified according to WHO growth charts [17] For preterm infants, age was corrected by gestational age at delivery and classified using the INTERGROWTH-21st growth charts [17]. Within this group, laboratory confirmation of prenatal ZIKV exposure was based on molecular testing for the presence of ZIKV RNA by qRT-PCR in a sample of cerebrospinal fluid or through serology for the detection of ZIKV- specific IgM antibodies using MAC-ELISA or based on the criteria of França, et al (2016) [20,21].
iii) Children with suspected prenatal ZIKV exposure who were referred to participating health services because they had other abnormalities consistent with congenital ZIKV infection, such as grossly abnormal clinical and/or brain imaging findings accompanied or not by neurological and ocular defects regardless of laboratory confirmation of prenatal ZIKV exposure, but after exclusion of other congenital infections [20,21].

Inclusion criteria encompassed laboratory-confirmed or suspected prenatal ZIKV exposure and normal neonatal hearing screening assessed via auditory brainstem response (ABR). Universal neonatal hearing screening (NHS) performed in the first year of life and were interpreted as normal by the presence of wave V in two consecutive recordings for each ear with an intensity of 35 dB nHL, like the international recommendations of the Joint Committee on Infant Hearing 2019 and the protocol published by Brazilian Ministery of Health. Exclusion criteria included failure in neonatal hearing screening with subsequent confirmation of sensorineural hearing loss (SNHL) upon diagnostic retesting, as well as incomplete data or absence of follow-up in auditory evaluations [22].

### Data

Data included information collected from the short latency auditory evoked potential of the brainstem with click stimullus (cABR) and behavioral observation audiometry (BOA) carried out by a qualified otorhinolaryngologist and speech therapist throughout evaluations [17]. The cABR was performed with a click-filtered stimulus (33–1500 Hz), stimulation rate of 37.7 milliseconds, rarefied polarity, and intensity of 35 dB nHL in the Vivosonic Integrity equipment. In case of cABR failure, the exam was repeated with an interval of up to one month (retest) and simultaneous performance of tympanometry to identify possible alterations in the middle ear, using the AT 235h equipment from Interacoustics (Vivosonic, Toronto, Canadá).

BOA was performed by presenting uncalibrated auditory stimuli to children using musical instruments, while observing their response to the sound. In this study, a rattle and drum were used, presented in free field under three progressively stronger intensities, as needed. During the assessment, the child remained alert, sitting with or without support, depending on the neurological condition and age. The sound stimuli were presented in the lateral plane, to the right and left and in the axial plane above and below eye level, lasting 2s, at an approximate distance of 30 cm from the ear [24].

Neurological assessments were performed by pediatric neurologists, including evaluations of consciousness, behavior, motor abnormalities, repeated head circumferences assessments, tonus or trophism, and pyramidal signs. Cognitive, motor and language development were assessed using the Bayley Scales of Infant and Child Development, Third Edition (B-III), a validated instrument for assessing child development between 1 and 42 months, which includes the cognitive, motor and language domains of neurodevelopment. For this research, only data from the composite language score were used, with a score below 85 points indicating a language delay [23].

### Follow-up

In this cohort, children were followed up every six months until they were 24 months old, and then annually, until they were 48 months old. During the periodic evaluations medical history, cABR, BOA and otorhinolaryngological physical examination were sistematically performed.

To monitor the evolution of auditory and language development in these children, we conducted three assessments at distinct age intervals. The first assessment was performed when the children were aged between 12 and 24 months old (second year of life), the second assessment when they were between 25 and 36 months old (third year of life), and the third and final assessment was conducted at 36 months or older (fourth year of life or beyond).

### Statistical analysis

Categorical variables were evaluated using Chi-squared and Fisher’s exact tests with 95% confidence intervals. Statistical analyses were performed using STATA/SE 12.0 and Excel 2019 software.

## Results

**Fig 1:**
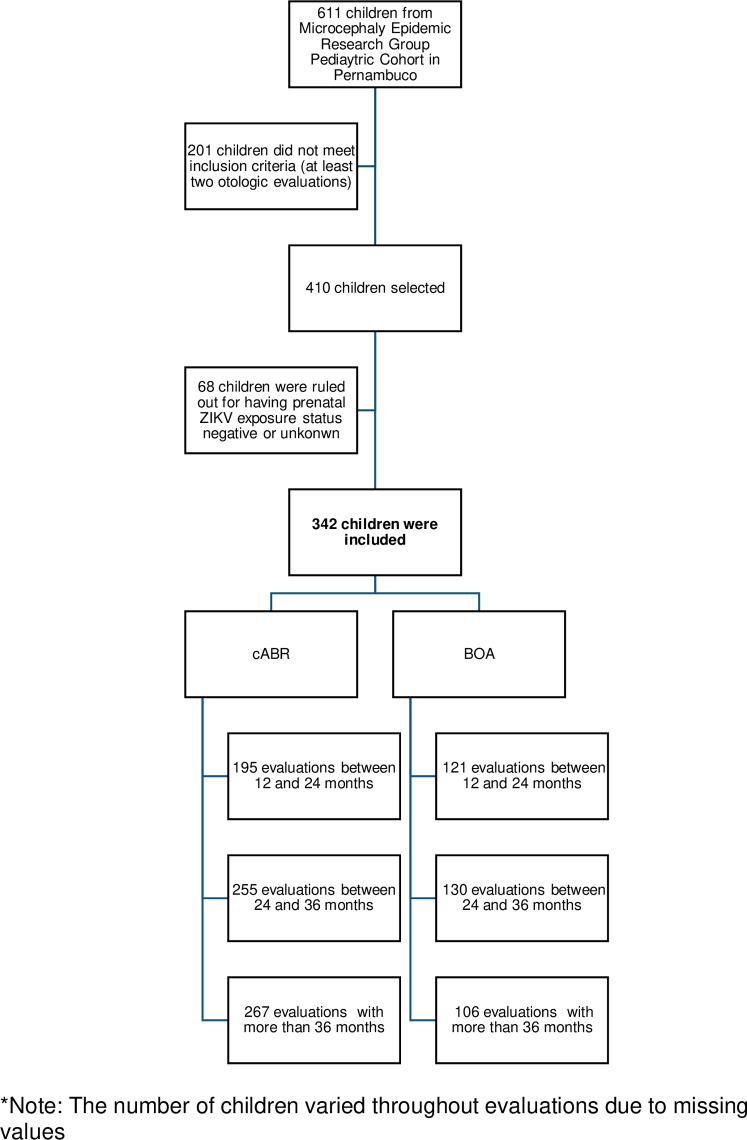
Participants flowchart. *Note: The number of children varied throughout evaluations due to missing values.

From the Microcephaly Epidemic Research Group Pediatric Cohort in Pernambuco [16], a total of 410 children who underwent two or more otologic evaluations were selected for this study. Of these, 68 were excluded for not meeting the eligibility criteria for prenatal ZIKV exposure. Consequently, 342 children with suspected or laboratory-confirmed prenatal ZIKV exposure were followed over the first four years of life. However, the number of children participating in each evaluation varied due to missed appointments, resulting in different sample sizes across assessments (Fig 1).

While 294 (73.2%) children had normal head circumference at birth, 108 (26.8%) had microcephaly, the majority in the severe form. Furthermore, 16 children born with microcephaly later achieved normal HCs, while 28 children born without microcephaly presented with late-onset microcephaly. Considering motor development, some characteristics were evaluated especially between 12 and 24 months of age, such as axial tone and cervical control. (Table 1)

**Table 1:** Characteristics of children with prenatal ZIKV exposure.

	Total n (%)	Sex		
		Female	Male	p-value
		n (%)	n (%)	
**Microcephaly**				
Born with microcephaly and developed normal HC	16 (4,0)	13 (81,2)	3 (18,8)	0,010 *
Born with microcephaly and remained with microcephaly	92 (22,9)	54 (58,7)	38 (41,3)	
Born without microcephaly and developed microcephaly	28 (7,0)	12 (42,9)	16 (57,1)	
Born without microcephaly and remained without microcephaly	266 (66,2)	122 (45,9)	144 (54,1)	
**Severity of microcephaly at birth**				
Mild (≤ - 2 SDs)	41 (39,4)	30 (73,2)	11 (26,8)	0,133 *
Severe (≤ - 3 SDs)	63 (60,6)	37 (58,7)	26 (41,3)	
**Cervical control 12–24 months**				
Stable	162 (83,1)	74 (45,7)	88 (54,3)	0,352 *
Unstable	33 (16,9)	18 (54,5)	15 (45,5)	
**Cervical control 24–36 months**				
Stable	140 (73,3)	66 (47,1)	74 (52,9)	0,478 *
Unstable	51 (26,7)	27 (52,9)	24 (47,1)	
**Cervical control > 36 months**				
Stable	8 (36,4)	6 (75,0)	2 (25,0)	0,649 **
Unstable	14 (63,6)	8 (57,1)	6 (42,9)	
**Axial tone 12–24 months**				
Normal	158 (81,0)	71 (44,9)	87 (55,1)	0,195 *
Altered	37 (19,0)	21 (56,8)	16 (43,2)	
**Axial tone 24–36 months**				
Normal	126 (66,0)	61 (48,4)	65 (51,6)	0,915 *
Altered	65 (34,0)	32 (49,2)	33 (50,8)	
**Axial tone > 36 months**				
Normal	6 (27,3)	4 (66,7)	2 (33,3)	1,000 **
Altered	16 (72,7)	10 (62,5)	6 (37,5)	

(*) Chi-Square (**) Fisher’s exact test

† note: The number of children varied througout evaluations due to missing values

**Fig 2:**
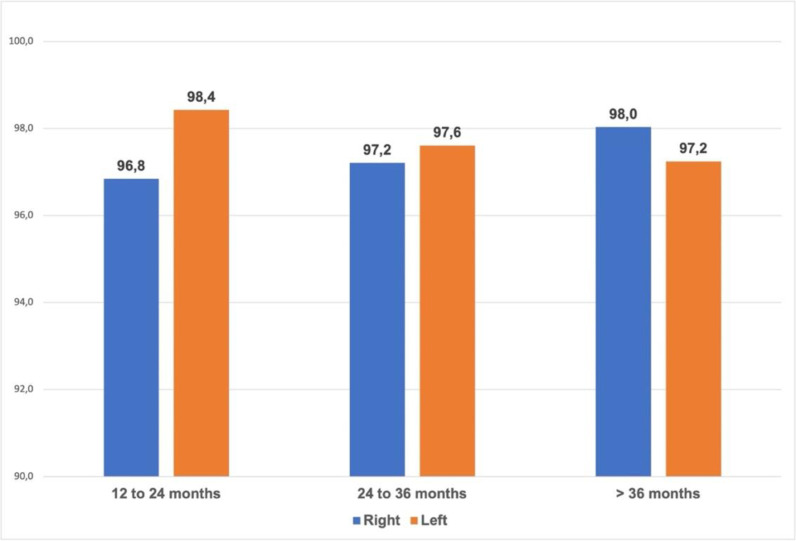
Auditory Brainstem Evoked Response (ABR).

Occasional failures in the three cABR evaluations were observed and attributable to the presence of middle ear effusion as these children demonstrated normal cABR thresholds upon retesting (Fig 2). This finding indicates that these transient auditory impairments were conductive in nature and not indicative of SNHL. Regarding the BOA, all children presented response of attention to sound in response to a low-intensity stimulus in the three assessments, except for a single child between 12 and 24 months.

Latency, defined as the time between the presentation of the stimulus and the child’s response, exceeded 2 seconds in 91.3% (310/340), 96.8% (285/294), and 100% (270/270) of children evaluated during the first (12–24 months), second (25–36 months), and third (≥37 months) assessments, respectively. Conversely, the direct and complete localization of the sound source was observed in 76.3% (260/340), 90.4% (266/294), and 96.2% (260/270) of children during the same evaluations (Table 2).

**Table 2:** Behavioral audiometry from the first to the fourth year in children with prenatal ZIKV exposure.

	Total n (%)	Sex		
		Female	Male	p-value
		n (%)	n (%)	
**Latency**				
**12 to 24 months**				
Normal	10 (8,7)	5 (50,0)	5 (50,0)	0,753 *
Altered	105 (91,3)	47 (44,8)	58 (55,2)	
**24 to 36 months**				
Normal	4 (3,2)	0 (0,0)	4 (100,0)	0,124 *
Altered	122 (96,8)	58 (47,5)	64 (52,5)	
**> 36 months**				
Normal	0 (0,0)	0 (0,0)	0 (0,0)	**
Altered	106 (100,0)	58 (54,7)	48 (45,3)	
**Attention to sound**				
**12 to 24 months**				
Present	117 (99,2)	52 (44,4)	65 (55,6)	0,449 *
Absent	1 (0,8)	1 (100,0)	0 (0,0)	
**24 to 36 months**				
Present	129 (100,0)	58 (45,0)	71 (55,0)	**
Absent	0 (0,0)	0 (0,0)	0 (0,0)	
**> 36 months**				
Present	105 (100,0)	57 (54,3)	48 (45,7)	**
Absent	0 (0,0)	0 (0,0)	0 (0,0)	
**Sound localization**				
**12 to 24 months**				
Complete	90 (76,3)	38 (42,2)	52 (57,8)	0,787 *
Incomplete	25 (21,2)	11 (44,0)	14 (56,0)	
Absent	3 (2,5)	2 (66,7)	1 (33,3)	
**24 to 36 months**				
Complete	113 (90,4)	51 (45,1)	62 (54,9)	0,466 *
Incomplete	7 (5,6)	4 (57,1)	3 (42,9)	
Absent	5 (4,0)	1 (20,0)	4 (80,0)	
**> 36 months**				
Complete	100 (96,1)	55 (55,0)	45 (45,0)	1,000 *
Incomplete	3 (2,9)	2 (66,7)	1 (33,3)	
Absent	1 (1,0)	1 (100,0)	0 (0,0)	

(*) Fisher’s exact test (**) not calculable

† note: The number of children varied througout evaluations due to missing values

The ability to completely localize the sound source was associated with the presence of a normal HC at birth (p-value < 0,05 in all age groups - Table 3). However, altered latency to the sound stimulus was more frequent in children without microcephaly. There was also a significant association between BOA alterations and body hypotonia and inadequate cervical control, especially in children younger than 3 years (Tables 3 and 4). In the first assessment (12–24 months) 23,7% of the children had language delay in the composite score of Bayley test, in the second assessment (24–36 months) 18% of the children had language delay in this composite score, while in the third assessment (> 36 months), 5,3% had language delay. In just two evaluations there was incomplete localization of the sound source, and it happened in children with adequated language score.

**Table 3:** Behavioral observational audiometry in children with prenatal ZIKV exposure according to the microcephaly status.

	Children with Microcephaly		Children without Microcephaly	p-value
	Mild n (%)	Severe n(%)	n(%)	
**12 a 24 months**				
**Latency**				
Normal	1 (7,1)	6 (25)	1 (1,4)	0,001 *
Altered	13 (92,8)	18 (75)	70 (98,5)	
**Sound Localization**				
Complete	10 (71,4)	11 (42,3)	64 (90,1)	< 0,001 *
Incomplete	4 (28,5)	13 (50)	7 (9,8)	
Absent	0 (0)	2 (7,6)	0 (0)	
**24 a 36 months**				
**Latency**				
Normal	0 (0)	3 (12)	0 (0)	0,010 *
Altered	16 (100)	22 (88)	81 (100)	
**Sound Localization**				
Complete	16 (100)	19 (73)	76 (96,2)	0,009 *
Incomplete	0 (0)	4 (15,3)	2 (2,5)	
Absent	0 (0)	3 (11,5)	1 (1,2)	
**>36 months**				
**1Latency**				
Normal	0 (0)	0 (0)	0 (0)	**
Altered	11 (100)	16 (100)	75 (100)	
**Sound Localization**				
Complete	10 (90,9)	14 (87,5)	72 (98,6)	0,031 *
Incomplete	0 (0)	2 (12,5)	1 (13,6)	
Absent	1 (9)	0 (0)	0 (0)	

(*) Fisher’s Exact test (**) Not Calculable

*Note: The number of children varied throughout evaluations due to missing values

#Note 2: p-values refers to comparison between children with microcephaly vs. children without microcephaly.

**Table 4:** Association between neurological findings and sound localization.

		Sound Localization		
Neurologial Assessment	Complete	Incomplete	Absent	p-value
	n (%)	n (%)	n (%)	
***12 a 24 months***				
**Posture**				
Appropriate	50 (98,0)	1 (2,0)	0 (0,0)	**< 0,001** *
Inappropriate	10 (35,7)	15 (53,6)	3 (10,7)	
**Neck control**				
Estable	53 (94,6)	3 (5,4)	0 (0,0)	**< 0,001** *
Unstable	7 (30,4)	13 (56,6)	3 (13,0)	
***24 a 36 months***				
**Posture**				
Appropriate	48 (100,0)	0 (0,0)	0 (0,0)	**< 0,001** *
Inappropriate	26 (74,3)	4 (11,4)	5 (14,3)	
**Neck control**				
Estable	54 (98,2)	1 (1,8)	0 (0,0)	**< 0,001** *
Unstable	19 (70,4)	3 (11,1)	5 (18,5)	
***>36 months***				
**Posture**				
Appropriate	3 (100,0)	0 (0,0)	0 (0,0)	1,000 *
Inappropriate	5 (71,4)	1 (14,3)	11 (14,3)	
**Neck control**				
Stable	3 (100,0)	0 (0,0)	0 (0,0)	1,000 *
Unstable	5 (71,4)	1 (14,3)	11 (14,3)	

(*)Fisher’s exact test

## Discussion

In this study, in children with or without microcephaly, who had evidence of exposure to ZIKV during fetal life, with no SNHL in the first year of life, the occurrence of late onset SNHL was not observed during auditory and language monitoring up to four years of life. Throughout the study period, only transient hearing losses, most often related to sound conduction disorders such as middle ear effusion, were identified. Therefore, the findings of this cohort suggest that late onset congenital SNHL does not seem to occur among infants and preschoolers exposed to ZIKV during fetal life. We also found a positive association between the incomplete or absent sound location and the severity of microcephaly through the behavioral observation audiometry.

To date, there is no prospective study with a follow-up of more than 3 years of children exposed to ZIKV during pregnancy without microcephaly. Moreover, there was no follow up focusing children with normal NHS. In our study, there is no evidence of late SNHL associated with ZIKV in a sample composed predominantly of children with normal head circumference, but with evidence of fetal exposure to ZIKV. In a previous study by our group involving a 3-year follow-up of 107 children with Zika-related microcephaly, also did not found cases of late-onset or progressive deficits in hearing [9].

When pure-tone audiometry is not possible in young children, a complete hearing assessment requires a combination of several tests, including electrophysiological tests and behavioral assessment in order to obtain an analysis of specific thresholds encompassing all frequencies in the 250–4000Hz [19]. Therefore, the assessment of the sound response through observational behavioral audiometry was associated with cABR in successive evaluations throughout the follow-up of the children in this cohort.

Moreover, althoug most children were able to completely locate the sound source, through BOA evaluation, the abnormal response was directily related to the severity of microcepahly in our sample, as 57% of children with severe microcephaly and 28% of children with mild microcephaly still had incomplete or absent localization of the sound source in the second year of life, while only 9% of children without microcephaly had some degree of limitation in locating the sound source. Indirect localization of the sound stimulus presented below eye level is expected to happen between 4 and 7 months old, whilst direct localization of the sound source happens between 9 and 11 months old and by the age of 12 months of life, children are expected to find an active interest in the environment with rapid localization of auditory signals with a minimum average threshold of 10 dB nHL [24]. A prior cross-sectional study using BOA in children with congenital ZIKV infection found an incomplete or absent localization of the sound source in nearly 74% of the children. The differences between this data and our results can be explained by the younger age average and the presence of microcephaly in more than 70% of the children analyzed in the referred cross-sectional study [15].

Although microcephaly seems to be related to the incidence of hearing loss, the severity of such malformation in our sample was inversely related to the latency response to the sound stimulus presented in the BOA, which possibly could have been an error in the examiner’s interpretation, having in view of the subjective character of the test and the risk of overestimating muscle spasms and myoclonus as a response to attention to sound in children with relevant neurological abnormalities, as in the case of ZIKV-related microcephaly. This hypothesis is supported by the statistically significant association between the ability to locate the sound source and the presence of a normal head circumference at birth, since this ability is hierarchically superior in children’s auditory development.

In addition, abnormalities in neuropsychomotor development, such as unstable neck control, inappropriate axial tone and posture were positivily associated with the child’s limitation to identify the location of the sound stimulus performed in the present study, since the tonus of the axial muscles is directly associated with the development of the motor ability to search for the sound source it is rationalized that congenital neurological abnormalities co-occurring with cervical hypo or hypertonia have the potential to impact language development as demonstrated in in a previous cross-sectional study [15,25,26,27]. However, despite the positive association between unstable neck control and incomplete or absent sound source localization found in our study population, another possible explanation for this altered response to sound stimullus could be the presence of neurobehavioral disorders that weren’t explored in our cohort [28,29,30,31,32].

In this study language assessment through the Bayley’s child neurodevelopment scale was adequated in most evaluations in children without microcephaly but we not observed association with sound location through BOA, as only two evaluations that showed incomplete localization of sound were observed in children with normal Bayley’s language composite score. Recent studies also demonstrated that children exposed to ZIKV without microcephaly do not present increased risk of neurodevelopmental impairment [13,14,32]. Although language assessment is much more complex than the auditory elements considered in this study, it is very likely that the presence of normal peripheral hearing at birth, without evidence of progressive impairment of auditory capacity over the first four years of life, in the absence of microcephaly, has contributed to language development as expected for age.

Behavioral observational auditometry used in this research is a subjective evaluation, whose interpretation is susceptible to intra and inter-observer variations over time, specially when neuropsychomotors disorders are present leading to misclassification. It may have been a limitation in our stydy, allowing random muscle reactions such as myoclonus to be interpreted as responses of attention to sound in some children. Another issue is the possibility to underestimate the sound location in the presence of unstable neck control and/or axial hypo/hypertonia that could limit head movement towards the sound source. To minimize this problem, we considered partial head or eye movements towards sound source as a positive response. It should be noted that there is no specific tool to evaluate neurologic impaired children’s response to sound. To minimize the subjectivity of BOA all procedures were standardized and cared out by just one examiner in our study.

Another possible limitation of our study is that the number of children evaluated through the longitudinal assessments varied due to losses of follow-up, but it is unlikely that selection bias occurred, since participants were continually reinvited for periodic assessments throughout the study period by multidisciplinary task forces.

A strong contribution of this research is the fact that this is the longest auditory follow-up of children exposed to ZIKV during the fetal period so far. In addition, this study used precise methods for assessing auditory thresholds, such as the ABR for screening and the frequency specific ABR for diagnostic confirmation, in addition to having a good sample of children with normal HC at birth (73.2%), contributing to clarifying aspects of congenital ZIKV infection that go beyond microcephaly.

## Conclusion

This study fills a gap in scientific knowledge regarding the auditory functions of children exposed to ZIKV during fetal life. It is possible to affirm, based on the presented audiological findings, that congenital sensorineural hearing loss caused by ZIKV has a typical presentation at birth, with no evidence so far of a late-onset or progresive presentation throughout the first years of life.

Furthermore, children with prenatal ZIKV exposure, without microcephaly and with normal neonatal hearing screening, do not have major repercussions on the behavioral response to sound and thus can be followed up according to the recommendations for the general population, not being at an apparently greater risk of damage to auditory development in childhood.
